# Carcinoid Heart Disease: The Role of Echocardiography in Raising the First Suspicion

**DOI:** 10.3390/jcm15051978

**Published:** 2026-03-05

**Authors:** Silvia Stavagna, Giovanna Manzi, Danilo Angotti, Andrea D’Amato, Elisa Giannetta, Roberto Badagliacca, Federico Ciccarelli, Lucrezia Netti, Paolo Severino, Wael Saade, Carmine Dario Vizza, Viviana Maestrini

**Affiliations:** 1 Department of Medical and Cardiovascular Sciences, Policlinico Universitario Umberto I, Sapienza University of Rome, 00161 Rome, Italy; stavagnas@gmail.com (S.S.); danilo.angotti95@gmail.com (D.A.); damatoandrea92@gmail.com (A.D.); roberto.badagliacca@uniroma1.it (R.B.); federico.ciccarelli@uniroma1.it (F.C.); lucrezia.netti@uniroma1.it (L.N.); paolo.severino@uniroma1.it (P.S.); dario.vizza@uniroma1.it (C.D.V.); viviana.maestrini@uniroma1.it (V.M.); 2 Department of Experimental Medicine, Sapienza University of Rome, Viale Regina Elena, 324, 00161 Rome, Italy; elisa.giannetta@uniroma1.it; 3 Department of Clinical, Internal Anesthesiological and Cardiovascular Sciences, Sapienza University of Rome, 00161 Rome, Italy; wael.saade@uniroma1.it

**Keywords:** carcinoid heart disease, carcinoid cardiopathy, echocardiography, transcatheter management

## Abstract

Neuroendocrine tumors (NETs) are rare neoplasms arising from the diffuse neuroendocrine system that can range from indolent to highly aggressive diseases. They usually clinically manifest when reaching a significant size or when hepatic metastases develop, leading to overproduction and impaired hepatic metabolism of vasoactive substances. The clinical course of NETs may be complicated by cardiac involvement, known as carcinoid heart disease (CHD), predominantly affecting the right side of the heart. CHD is characterized by specific echocardiographic features, including thickening, reduced excursion and retraction of valvular leaflets, resulting in valvular stenosis or regurgitation. Despite its clinical relevance, awareness of CHD as a complex hormonal sequela of NETs remains limited among cardiologists, and its echocardiographic findings are not universally recognized. This review aims to (a) provide cardiologists with the main principles for understanding CHD pathophysiology; (b) illustrate the main echocardiographic features of CHD, using a stepwise approach; and (c) refine a diagnostic algorithm for detecting cardiac involvement in NET populations and identifying patients at high risk of developing CHD.

## 1. Introduction

Neuroendocrine tumors (NETs) represent a heterogeneous group of rare neoplasms arising from enterochromaffin cells. They can originate in multiple anatomical locations, including the head and neck, thymus, thyroid, breasts, skin and the genitourinary system, but the gastrointestinal tract and the lungs are the most common primary sites.

Epidemiological analyses from the Surveillance, Epidemiology, and End Results (SEER) registries in the United States report a 5.2-fold rise in age-adjusted incidence of NETs between 1975 and 2021 [1]. The increasing incidence observed worldwide over recent decades may be attributed to improved disease awareness, enhanced detection and advances in diagnostic techniques.

Worldwide, several scientific societies are engaged in developing an NET registry, a crucial database that systematically collects patient data on diagnosis, tumor stage, treatment and outcomes. The most known include the multinational ENETS Registry (European Neuroendocrine Tumor Society) and national ones like Italy’s Itanet and Spain’s RGETNE [2]. These registries help clinicians in understanding NET epidemiology, improving standards of care, and guiding research. Nevertheless, challenges exist in data standardization and completeness across registries. Patient-centered models have emerged to overcome these issues, such as the PLANET Registry Australia, which directly involves patients [3] in data collection by capturing information related to quality of life and daily parameters.

Since 2008, ENETS has maintained an NEN registry. In 2020, ENETS partnered with the Coordinating Center for Clinical Trials at the Philipps-University of Marburg (KKS) to elevate the project to new academic levels. A key objective of this overhaul was to ensure the integrity and reliability of the data collected, providing more robust and accurate insights.

### 1.1. NET Classification

NETs can be classified as “functional” (around 10–40%) or “non-functional” (approximately 60–90%) based on their secretory profile. Functional NETs release bioactive amines into the bloodstream, most notably serotonin, tachykinins, histamine, prostaglandins and kallikrein. Among patients with functional NETs, approximately 19% develop a carcinoid syndrome (CS), a clinical condition characterized by chronic diarrhea and/or flushing in the presence of elevated systemic levels of serotonin or its metabolite, 5-hydroxyindolacetic acid (5-HIAA) [4]. Importantly, alternative causes of these symptoms should be considered and investigated depending on the clinical presentation.

CS is predominantly encountered in patients with well-differentiated grade 1 or grade 2 NETs, most commonly arising from the intestine, followed by lung NETs. It is rarely reported in patients with pancreatic, ovarian, thymic, or NETs of unknown origin, named UKO. The hallmark symptoms of CS include skin flushing, secretory diarrhea, bronchospasm, and, in some cases of advanced intestinal NETs, abdominal pain, in the context of systemically elevated levels of serotonin and/or other biologically active amins and peptides.

Patients with CS suffer from an impaired quality of life, which is lower when compared to patients without CS or other types of cancer [5,6]. Furthermore, nearly half of patients with CS subsequently develop carcinoid heart disease (CHD), a major determinant of morbidity and mortality.

### 1.2. Carcinoid Heart Disease: The Pathophysiology

The development of CHD is typically associated with advanced NETs and/or metastatic involvement of the liver, as vasoactive compounds secreted by NETs bypass hepatic inactivation and reach the right side of the heart. Additionally, liver or retroperitoneal metastases may continuously produce serotonin (5-hydroxytryptamine, 5-HT), which is directly released into circulation at high levels. CHD predominantly affects right-sided cardiac valves, whereas left-sided valvular or chamber involvement is rare—occurring in approximately one-third of cases—since serotonin and other humoral substances are usually inactivated within the pulmonary circulation. The presence of left-sided CHD should prompt clinicians to investigate a patent foramen ovale (PFO). Indeed, this tunnel-like interatrial communication, which may open in response to increased right atrial pressure and volume, allows vasoactive substances to bypass pulmonary deactivation and enter the systemic circulation, ultimately causing mitral or aortic regurgitation. The prevalence of PFO in patients with CHD is higher than that in the general population (approximately 59% vs. 25%) [7]. Less commonly, left-sided CHD may occur in patients with primary bronchial endocrine tumors or in the presence of very high levels of circulating serotonin which may exceed the lung’s degradative capacity.

Although not fully understood, the pathophysiology of CHD is closely related to chronic exposure of the endocardium to circulating vasoactive substances with fibroblast proliferative properties, particularly 5-HT. Serotonin, derived from the essential amino acid tryptophan, acts through receptors that are well represented in the heart, supporting serotonin’s central role in CHD development. The resulting pathological process leads to the formation of plaque-like fibrotic deposits that may involve the endocardial surfaces of valve leaflets, the subvalvular apparatus, and the cardiac chambers [8]. These lesions are composed of myofibroblasts, smooth muscle cells, and extracellular matrix components, including collagen, myxoid matrix, and elastin.

### 1.3. Biomarkers of CHD

Serotonin or its main metabolite 5-hydroxyindoleacetic acid (5-HIAA) should be measured at presentation in all patients with advanced intestinal NETs, in lung/ovary NETs of any stage, in unknown origin (UKO) NETs with liver metastases, and in every NET patient with suspected CS. Whole blood serotonin assay is limited by its saturation in platelets at 40 nmol/10^9^ platelets, making it less suitable for CS monitoring. So, a 24 h urinary 5-HIAA (u5-HIAA) secretion above 50 μmol is considered compatible with the diagnosis of CS (u5-HIAA normal range: 2 to 9 mg/24 h or 10.4 to 46.8 μmol/24 h). u5-HIAA is significantly higher in patients with than those without CHD, and its peak level is a significant predictor of progressive CHD. u5-HIAA > 300 μmol confers a two- to three-fold increase in the risk of CHD development/progression. The primary screening of u5-HIAA as a tumor marker is limited by incorrect sampling, serotonin-rich food, or serotoninergic drug intake, leading to false-positive results. Because of significant variability, u5-HIAA should be preferably assessed in two 24 h urine collections (the ratio of u-5HIAA/creatinine in a spot urine may be used for follow-up of patients with known elevated u5-HIAA levels).

Plasma 5-HIAA is an alternative method effectively differentiating CS patients from controls and correlating with the presence of CS and CHD.

Chromogranin A (CgA) has been suggested as a relevant marker for CS and CHD, but levels do not differentiate between NET patients with or without CS [5,9,10].

In addition, N-terminal pro-B-type natriuretic peptide (NT-proBNP), traditionally used as a diagnostic biomarker for heart failure, may serve as a predictor of CHD. In this regard, Bhattacharyya et al. conducted a prospective study enrolling 204 patients with carcinoid tumors of midgut origin and a history of carcinoid syndrome. CHD was present in 39 patients (19.5%). Median NT-proBNP levels were significantly higher in patients with CHD than in those without CHD (1.149 pg/mL vs. 101 pg/mL respectively, *p* < 0.001). The authors found that NT-proBNP with a cut-off level of 260 pg/mL (31 pmol/L) may aid clinicians in screening patients with carcinoid syndrome for the presence of CHD [11].

It should be noted that the circulating concentration of NT-proBNP is influenced by several cardiac conditions, such as atrial fibrillation and heart failure, as well as by non-cardiac factors, including age, renal function, and obesity. Therefore, defining a universal threshold value to distinguish patients with carcinoid heart disease is challenging. NT-proBNP levels should be interpreted within the individual context, taking into account patient characteristics, and integrated into a comprehensive clinical, laboratory and imaging assessment.

### 1.4. Carcinoid Heart Disease: Why Cardiologists Should Care

A deeper understanding of CHD is relevant for patient outcomes and has important implications for clinical practice, both for endocrinologists and cardiologists. Indeed, referral for cardiac surgery without recognition of the underlying NET may lead to harmful therapeutic decisions. Carcinoid crisis (CC)—a severe life-threatening complication characterized by bronchospasm, intense flushing, and severe hemodynamic instability—may occur during tumor manipulation, administration of sympathomimetic drugs, anesthesia, and cytolytic therapies such as hepatic embolization. To prevent peri-operative CC, the 2022 ESC Guidelines on cardio-oncology [12] recommend starting the infusion of intravenous somatostatin analogs (e.g., octreotide) on the morning of the procedure (up to 12 h preoperatively). Treatment should be continued throughout the intervention (including surgery, preoperative coronary angiography, or pacemaker implantation) and maintained postoperatively for at least 48 h following valve surgery or until clinical stability is achieved.

Nevertheless, awareness of CHD as a complex hormonal sequela of NETs remains limited among cardiologists, and its echocardiographic features are not universally recognized. This review aims to (a) provide a comprehensive overview of the echocardiographic features of CHD; (b) briefly discuss current medical and surgical management strategies; and (c) refine a practical surveillance algorithm for the screening of patients with NETs and CS.

## 2. Materials and Methods

A comprehensive literature search for this narrative review was conducted on the electronic databases PubMed, Embase and Cochrane. The search strategy employed the following keywords: “carcinoid cardiopathy” OR “carcinoid heart disease” AND “echocardiography”. Studies were included if they focused on the role of echocardiography in the diagnosis of CHD and provided original data obtained using rigorous methodological approaches. Studies published up to December 2025 were considered, with inclusion limited to articles published in English.

## 3. The Role of Echocardiography and Cardiac Imaging in CHD

Transthoracic echocardiography represents the cornerstone imaging modality for the diagnosis of CHD. In patients with NETs, a comprehensive echocardiographic assessment should include:

1. **Detailed evaluation of cardiac valves**, with focus on the tricuspid and pulmonary valves.
2. **Assessment of right-sided cardiac chamber size and function**.
3. **Evaluation for the presence of a PFO**, particularly in patients with left-sided carcinoid heart disease and/or to identify those at high risk of CHD progression.
4. **Screening for myocardial metastases**.
5. **Calculation of disease-specific score systems**.

1. **Detailed evaluation of cardiac valves.** Due to plaque-like fibrotic deposits, the valves become thickened, retracted and fixed, ultimately resulting in regurgitation and/or stenosis. The tricuspid valve is involved in up to 90% of cases, followed by the pulmonary (69%), mitral (29%), and aortic (27%) valves [13].
  In advanced cases, the tricuspid valve has a dilated annulus and extended thickened and retracted leaflets that fail to coapt during systole, resulting in a central gap and severe tricuspid regurgitation. On continuous-wave Doppler, the regurgitant signal appears densely saturated and, rather than exhibiting a parabolic contour, displays an early-peaking triangular configuration (often described as “dagger-shaped”). The peak regurgitant velocity is usually low (<2 m/s), suggesting that right ventricular systolic pressures are not elevated as in pulmonary hypertension. As a result, the right ventricle (RV) and the right atrium (RA) functionally behave as a single chamber.
  In the context of CHD, tricuspid stenosis is uncommon. According to the ENETS Carcinoid Heart Disease Task Force, its severity can be graded based on the tricuspid mean transvalvular gradient as mild (<5 mmHg), moderate (5–8 mmHg) and severe (>8 mmHg) [14].
  As observed for the tricuspid valve, pulmonary valve leaflets in CHD appear thickened and retracted and have reduced mobility, resulting in pulmonary regurgitation and/or stenosis. However, echocardiographic assessment of the pulmonary valve and accurate quantification of its regurgitation in CHD are challenging because of limited acoustic accessibility. Parasternal and subcostal right ventricular outflow tract views should, therefore, be carefully examined. Moreover, in patients with torrential tricuspid regurgitation, the pulmonary valve pathology (PV) is often masked, as both stenotic gradients and regurgitant volumes may be underestimated due to the reduced forward stroke volume reaching the PV. Thus, in this setting, the most informative parameter is the continuous-wave Doppler profile: a regurgitant jet ending before the onset of the next forward flow signal is suggestive of severe pulmonary regurgitation.
  The subcostal four-chamber view may be helpful for assessing RV free wall thickness, as the presence of RV hypertrophy may suggest pulmonary stenosis. Given the listed echocardiographic limitations, patients with CHD who are candidates for tricuspid valve replacement require a more accurate assessment of pulmonary valve pathology through cardiac magnetic resonance (CMR) [15].
2. **Assessment of right-sided cardiac chamber size and function.** The significant volume overload imposed by tricuspid and pulmonary regurgitation results in RV chamber dilation and diastolic ventricular septal flattening. Firstly, the RV preserves cardiac output thanks to compensatory mechanisms, which ultimately fail. The evaluation of right-sided cardiac chamber size and function includes tricuspid annular plane systolic excursion (TAPSE), systolic peak longitudinal tissue Doppler velocity at the RV free wall base (S′), RV fractional area change (FAC), three-dimensional ejection fraction (3D EF) and RV longitudinal strain. The following echocardiographic parameters are suggestive of RV dysfunction: TAPSE < 17 mm, RV TDI s’ < 10 cm/s, RV FAC ≤ 35%, 3D RV EF < 50%, RV free wall strain < 23% and RV global longitudinal strain < 21% [16].
  Of note, RV strain is reduced in patients with CHD, even when conventional systolic function parameters are initially preserved, and may be mildly impaired even before overt cardiac involvement in patients with elevated 5-hydroxyindoleacetic acid (5-HIAA) levels. However, a retrospective observational study by Alabdaljabar et al. [17], including 138 patients with confirmed CHD, demonstrated that reduced RV strain was not associated with increased mortality during a median follow-up of 5.0 years. In contrast, reduced right atrial (RA) reservoir strain emerged as an independent predictor of congestive heart failure development. These findings support the role of myocardial strain analysis in the early detection of cardiac involvement in patients with CS [18] and highlight the prognostic role of RA reservoir strain in reflecting disease-related morbidity in advanced CHD. Although echocardiography remains the first-line imaging modality, CMR is more accurate and highly reproducible in the assessment of RV volumes and function.
3. **Evaluation for the presence of a PFO.** PFO should be systematically assessed by contrast-enhanced transthoracic echocardiography in patients with left-sided carcinoid heart disease and/or to identify those at high risk of CHD progression. The exam consists of the injection of an agitated 5% glucose solution through an upper-extremity vein. A PFO is detected when contrast appears in the left atrium within the first three cardiac cycles, at rest, and after cough test or Valsalva maneuver. In patients who are candidates for percutaneous PFO closure, transesophageal echocardiography is required for preprocedural imaging assessment and device size selection.
4. **Cardiac metastases** in patients with CHD are uncommon (3.8%) and are usually located in the RA. Echocardiography may suggest their presence through deformation of the endocardial contour, but cardiac MRI is usually required for better characterization, including precise determination of lesion location, number, size, and spatial relationship to cardiac anatomical structures. Myocardial metastases have a high T2-weighted signal and isointense T1-weighted signal with postcontrast perfusion. Positron emission tomography (PET) represents an alternative imaging modality to CMR in the detection of metastatic lesions by showing their focal uptake.
5. **Several echocardiographic scoring systems** have been proposed to quantify the severity of CHD. The most comprehensive one was developed by Bhattacharyya S. et al., a 66-point assessment including evaluation and grading of the following echocardiographic characteristics: leaflet thickening, mobility, morphology, valvular stenosis, valvular regurgitation of all four valves, and RV diameter and function [19]. The scoring systems currently available in this field were compared by Dobson and colleagues in a prospective study enrolling 100 patients with metastatic NETs (21 with CHD) [20]. The five scoring systems were calculated in each patient. All resulted in discrimination between patients with and without CHD and all correlated with NT-proBNP and plasma 5-HIAA levels.

**Figure 1:**
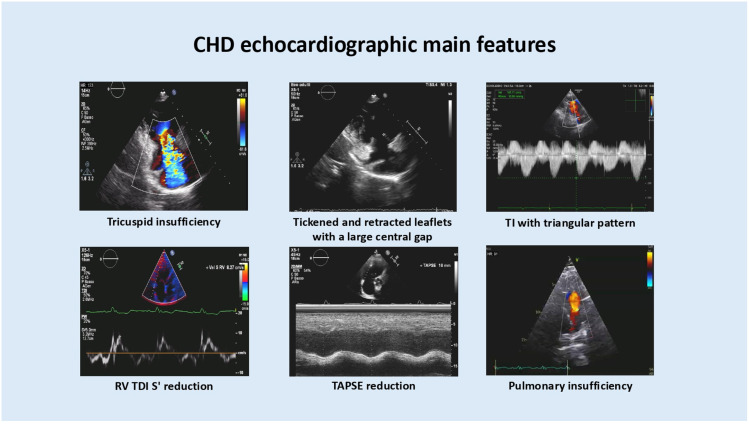
Main echocardiographic features observed in patients with carcinoid heart disease.

Figure 1 summarizes the main echocardiographic features of CHD.

## 4. Treatment of Carcinoid Heart Disease

A multidisciplinary approach involving neuroendocrine tumor oncologists, cardiologists, cardiac surgeons, endocrinologists and anesthesiologists is essential for the management of NETs. A full discussion of NET-specific therapies is beyond the scope of this review; however, the main therapeutic options are briefly outlined below.

Therapeutic strategies include antitumor treatment, prevention and control of CC, and targeted management of CHD.

Surgical intervention should be considered when both the primary NET and its associated metastases are amenable to complete resection. Conversely, first-line treatment with somatostatin analogs should be considered for patients with unresectable advanced NETs and favorable prognostic features. In patients with disease progression or poor prognosis, therapeutic options include chemoembolization for hepatic lesions, peptide receptor radionuclide therapy (PRRT), and Everolimus. Chemotherapy is generally reserved for patients with rapidly progressive metastatic diseases or failure of other treatments.

**A.**  **Surgical and Transcatheter Management of CHD: which approach and when?**

The surgical approach represents the standard of care for patients with CHD and severe valvular involvement and follows the recommendations of the 2025 ESC/EACTS guidelines for the management of valvular heart disease [16]. As previously illustrated, tricuspid regurgitation is the predominant valvular lesion. When surgery is indicated, valve repair is generally preferred over valve replacement; however, due to the characteristic structural alterations of the valvular apparatus, repair is often not technically feasible in this population. Of course, tricuspid valve replacement is usually the treatment of choice. During the same procedure, the pulmonary valve may also be addressed if indicated.

The selection of the valve prosthesis remains a matter of debate. In this setting, the multidisciplinary team plays a key role in determining the optimal timing of surgery and guiding prosthesis selection. Before the introduction of synthetic somatostatin and hepatic artery interruption, biological prostheses were not considered the first choice because of concerns regarding damage from vasoactive substances and accelerated degeneration. Conversely, mechanical prostheses require lifelong mandatory therapeutic anticoagulation, with consequent increased bleeding risk in patients with liver metastases [21]. Therefore, as a general rule, prosthesis selection in CHD should always be individualized, taking into account the patient’s overall life expectancy related to the underlying disease, the bleeding risk, and the likelihood of future interventions.

Although perioperative mortality has decreased in recent years, surgical treatment may still be complicated by severe events such as marked vasoplegia, carcinoid crisis and perioperative coagulopathy. Postoperative complications also include atrioventricular block, necessitating permanent pacemaker implantation in up to 25% of patients [22]. In many cases, right ventricular dysfunction persists despite tricuspid valve replacement.

Patients deemed at high or prohibitive surgical risk can be evaluated for transcatheter therapies, including the transcatheter tricuspid valve replacement (TTVR) with prostheses specifically designed for orthotopic implantation in the tricuspid position [23]. The anatomical characteristics of carcinoid tricuspid valves pose substantial challenges for transcatheter edge-to-edge repair, as the fibrotic process leading to thickened, retracted and fixed leaflets with a large gap precludes effective leaflet coaptation. However, these same pathological features make the disease more amenable to transcatheter orthotopic valve replacement, which does not rely on leaflet mobility for procedural success.

Given the high complexity of patients with CHD, management should be restricted to experienced centers with dedicated multidisciplinary teams—including cardiology, cardiac surgery, endocrinology, and anesthesiology—capable of coordinating pre- and post-procedural care and managing any sudden hemodynamic instability that may occur during transcatheter procedures, including the rapid deployment of mechanical circulatory support. Both team expertise and operator experience in optimizing patient preparation and promptly managing carcinoid crises are crucial to minimize perioperative risk and improve clinical outcomes.

**B.**  **Transcatheter Orthotopic Tricuspid Valve Replacement**

Currently, the only device to have received CE Mark approval is the EVOQUE valve, a transfemoral transcatheter system consisting of a self-expanding nitinol frame, a trileaflet bovine pericardial tissue valve and a fabric skirt designed to minimize paravalvular leak [24]. To date, only four cases of carcinoid-related tricuspid valve disease treated with TTVR have been reported in the literature, including two using the EVOQUE systems, one the LuX-Valve Plus system, and one an unspecified prosthesis. All patients were considered at high surgical risk.

The first attempt at transcatheter treatment of both tricuspid and pulmonary valve disease in a patient with CS was described by Pereyra et al. in Arizona [25]. The authors reported the case of a 79-year-old patient with CS from a small-bowel NET who complained of progressive exertional dyspnea. Transthoracic echocardiography revealed a severely enlarged RV with reduced systolic function, combined with severe pulmonary and tricuspid valve regurgitation. Both valves showed thickened, retracted, and fixed leaflets, findings typical of CHD. Given the high surgical risk, the patient underwent staged transcatheter replacement. The first procedure involved the pulmonary valve, with the use of a pre-stent into the RV outflow tract and a 29 mm S3 Sapien bioprosthesis implanted within the scaffold. However, heart failure symptoms persisted, and six months later, the patient underwent TTVR. The authors did not specify the device employed. The procedure was well tolerated, and the patient was discharged without complications. At 3-week follow-up, a significant improvement in symptoms was reported.

Recently, Dannenber et al. [26] reported the first successful combined pulmonary and tricuspid valve procedure in a 66-year-old patient with a previous diagnosis of a primary metastasized midgut NET, referred from the oncology department because of progressive dyspnea. Echocardiography revealed torrential tricuspid regurgitation and severe pulmonary regurgitation. Despite his relatively young age, the patient had significant comorbidities, with a EuroSCORE II of 14.36%. After careful pre-procedural planning, a 32 mm Venus *p*-Valve was implanted in the pulmonary position, followed by placement of a 44 mm EVOQUE bioprosthesis in the tricuspid position during the same procedure. Post-procedure transthoracic echocardiography demonstrated excellent function of both prostheses, with negligible residual regurgitation. The patient had no complications during recovery.

During the New York Valves meeting in 2025, Assafin et al. [27] presented a third case of a 77-year-old patient with hypertension, hyperlipidemia, stage III chronic kidney disease, and a metastatic carcinoid tumor, who was scheduled for abdominal surgical resection. The patient had severe tricuspid regurgitation, conferring an unacceptably high risk of postoperative bleeding following liver resection. After transesophageal echocardiography and CT evaluation, he was deemed a suitable candidate for TTVR and underwent implantation of a 44 mm EVOQUE valve, preceded by octreotide prophylaxis. Immediately after valve deployment, hemodynamics was favorable, with no residual tricuspid regurgitation and no evidence of pericardial effusion. However, after extubation, the patient developed profound hypotension and tachycardia, which progressed to cardiac arrest, requiring cardiopulmonary resuscitation and ECMO support. Transesophageal echocardiography confirmed the correct placement of the prosthesis and absence of pericardial effusion but revealed new onset of severe biventricular dysfunction. Massive pulmonary embolism, acute right-ventricular afterload mismatch, or a severe serotonin crisis were supposed as potential causes of the abrupt hemodynamic deterioration, which ultimately led to the patient’s death.

In conclusion, Veas et al. [28] reported the most recently published case of a middle-aged patient with a history of CS, recurrent ileal NET with liver metastases previously treated surgically, and stage IV chronic kidney disease. Echocardiography revealed thickened and restricted pulmonary and tricuspid leaflets, with severe pulmonary and torrential tricuspid regurgitation, without left heart involvement. Given the high surgical risk (TRI-SCORE of 7/12, corresponding to a predicted in-hospital mortality of 34%), the patient was selected for transcatheter valve intervention. Therefore, he underwent transcatheter pulmonary valve replacement (TPVR) with a Harmony 25 valve (Medtronic) and TTVR with the LuX-Valve Plus 30–40 system (Jenscare Scientific), preceded by subcutaneous administration of octreotide to prevent carcinoid crisis. Both prostheses were successfully implanted, and a reduction in the N-terminal pro–B-type natriuretic peptide levels was observed. In the following weeks, the patient developed systemic inflammatory response syndrome with acute kidney, hepatic and pulmonary failure, and died 25 days after the transcatheter procedure. Tumor progression, detected by PET/CT performed after TTVR, was suggested as a contributing factor to the patient’s outcome.

These interesting cases highlight that transcatheter valve intervention represents a promising therapeutic option for CHD patients at high or prohibitive surgical risk. However, further studies are needed to evaluate procedural safety, define the optimal selection of suitable candidates, and assess long-term clinical outcomes in this unique patient population.

### Heterotopic Transcatheter Tricuspid Valve Replacement

Heterotopic TTVR, consisting of valve implantation in the venae cavae, represents an alternative strategy for patients who are not suitable for either edge-to-edge repair or orthotopic TTVR. In this regard, Stoltz et al. [29] reported the case of a 70-year-old patient at high surgical risk whose anatomical characteristics precluded both leaflet repair, due to a large coaptation gap, and orthotopic TTVR, owing to excessive annular dilatation. Consequently, a heterotopic approach using the TricValve (P&F) system was selected. The procedure was successfully completed. At the 6-month follow-up, the patient reported marked symptomatic improvement, with resolution of peripheral edema and ascites, without RV functional deterioration.

Although clinically effective in patients with preserved or near-normal RV function, heterotopic TTVR should be regarded as a palliative approach, as it reduces systemic venous congestion without correcting the native tricuspid valve. Limitations include the risk of valve migration in the setting of severe RA dilatation, related to the complex geometry of the cavo-atrial junction. Moreover, after caval valve implantation, RA pressure and volume increase and may induce adverse RV remodeling [30].

**C.**  **Treatment of cardiac metastases**

To conclude the treatment section, a final remark concerns resection of cardiac metastases, which may be considered in patients with surgical indications for valve replacement. Conversely, isolated surgery for non-obstructive cardiac lesions is not recommended.

## 5. Discussion

CHD represents a major determinant of morbidity and mortality in patients with NETs. Despite increasing awareness and advances in oncological therapies, CHD is often diagnosed late, when right-sided valvular damage and RV dysfunction are already established.

Early identification of cardiac involvement is critical for improving patients’ prognosis. Transthoracic echocardiography plays a pivotal role in this setting, in both diagnosis and follow-up. Two-dimensional imaging is generally sufficient to identify the main disease manifestations, including valvular thickening and dysfunction, while earlier detection of cardiac involvement may be possible through strain analysis.

Nevertheless, echocardiography is not routinely performed in all patients with NETs. A combined screening strategy, integrating biochemical markers (such as urinary 5-HIAA and NT-proBNP) with echocardiography, may be the most effective approach for the early detection of CHD.

Accurate measurement of urinary 5-HIAA is critical for this strategy. Patients should collect all urine produced over 24 h and should be instructed to follow specific dietary and pharmacological restrictions for 3 days prior to urinary collection. Indeed, consuming foods rich in serotonin or tryptophan (e.g., bananas, pineapples, tomatoes, walnuts, avocados) within 24–72 h prior to the test can falsely raise urinary 5-HIAA levels. Similarly, certain medications (e.g., acetaminophen, cough syrup, antidepressants) may either increase or decrease urinary 5-HIAA.

**Figure 2:**
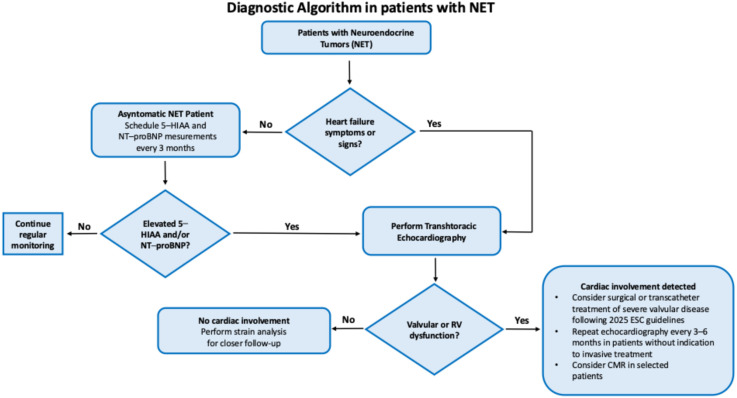
Diagnostic algorithm for the screening and management of cardiac involvement in patients with NETs.

We refined a diagnostic algorithm previously reported by Bober B. et al. [31] to enhance early detection of cardiac involvement in patients with NETs and to identify patients at high risk of developing CHD (Figure 2). Its validation is warranted in future prospective studies with clinically meaningful endpoints, such as overall survival and CHD-related mortality. In addition, stage of CHD at the time of surgery, peri-operative mortality in patients undergoing valve surgery, heart failure-related hospitalizations and quality of life measures should be assessed to capture the clinical impact of early detection of CHD.

Once an NET is diagnosed, two diagnostic pathways are possible, based on the presence of cardiac symptoms (e.g., dyspnea, shortness of breath) or signs suggestive of RV failure (e.g., ascites, peripheral edema).

1. Asymptomatic patients should undergo biomarker monitoring (urinary 5-HIAA and NT-proBNP) every three months. Patients with high or borderline levels should receive echocardiographic screening.
  (a) If no clear findings of CHD are detected, right-sided strain assessment is suggested to exclude early cardiac involvement.
  (b) If echocardiography screening confirms cardiac involvement, the exam should be repeated every 3–6 months, or sooner if signs of heart failure develop, to closely monitor disease progression. In patients with severe valvular disease, a surgical or transcatheter intervention should be considered, following the current ESC guideline recommendations.
2. Symptomatic patients should promptly receive a transthoracic echocardiography to assess for CHD.

Echocardiography is also recommended for all patients with metastatic NETs and for patients with CS.

**Figure 3:**
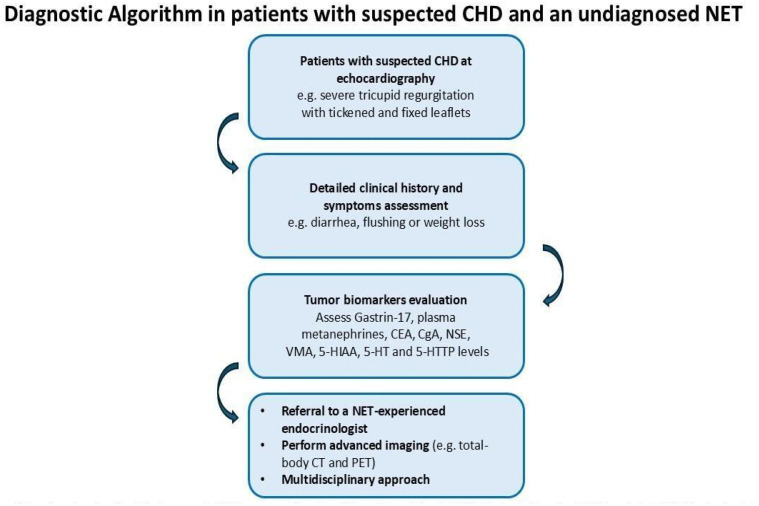
Diagnostic algorithm for patients with suspected carcinoid heart disease and an undiagnosed NET. CEA: carcinoembryonic; CgA: chromogranin A; NSE: neuron-specific enolase; VMA: vanillylmandelic acid; 5-HIAA: 5-hydroxyindoleacetic acid; 5-HT: serotonin; 5-HTTP: 5-hydroxytryptophan.

In other cases, the cardiologist may be the first to raise the suspicion of an NET based on characteristic echocardiographic findings. In such cases, a detailed clinical history should be obtained, combined with a comprehensive evaluation of tumor markers and advanced imaging tests (e.g., total-body CT scan). Referral to a multidisciplinary team with expertise in NETs is recommended (Figure 3).

## 6. Conclusions

Management of CHD requires a multidisciplinary approach focused on tumor control, prevention of carcinoid crisis, and timely treatment of valvular disease. Surgical replacement remains the standard of care for patients with severe symptomatic valvular disease and acceptable surgical risk. Nevertheless, transcatheter therapies are emerging as potential alternatives for patients with NETs and high surgical risk. Edge-to-edge repair is often unfeasible because of leaflet retraction and immobility, hallmarks of CHD. Conversely, orthotopic TTVR appears anatomically well suited in this setting. However, current evidence is limited to isolated case reports with heterogeneous outcomes, highlighting the need for cautious patient selection and comprehensive pre-procedural assessment. Heterotopic caval valve implantation may offer symptomatic relief in selected patients, but it should be regarded as a palliative strategy. A comprehensive, multimodal approach integrating clinical evaluation, biochemical markers, and imaging tools allows identification of patients at high risk of developing CHD, facilitates early recognition of cardiac involvement in patients with NETs and helps to prevent the progression to irreversible RV dysfunction.

## Data Availability

No new data were created or analyzed in this study. Data sharing is not applicable to this article.

## Abbreviations

The following abbreviations are used in this manuscript:

NETs	Neuroendocrine tumors
NENs	Neuroendocrine neoplasms
SEER	Epidemiological analyses from the Surveillance, Epidemiology, and End Results
ENETS	European Neuroendocrine Tumour Society
CHD	Carcinoid heart disease
5-HIAA	5-hydroxyindolacetic acid
CS	Carcinoid syndrome
NT-proBNP	N-terminal pro-B-type natriuretic peptide
5-HT	5-hydroxytryptamine
PFO	Patent foramen ovale
UKO	Unknown origin
CgA	Chromogranin A
u5-HIAA	Urinary 5-hydroxyindolacetic acid
ESC	European Society of Cardiology
RV	Right ventricle
RA	Right atrium
PV	Pulmonary valve
CMR	Cardiac magnetic resonance
TAPSE	Tricuspid annular plane systolic excursion
FAC	Fractional area change
PET	Positron emission tomography
PRRT	Peptide receptor radionuclide therapy
TTVR	Transcatheter tricuspid valve replacement
TPVR	Transcatheter pulmonary valve replacement
